# The need for pragmatic clinical trials in low and middle income settings – taking essential neonatal interventions delivered as part of inpatient care as an illustrative example

**DOI:** 10.1186/s12916-016-0556-z

**Published:** 2016-01-18

**Authors:** Mike English, Jamlick Karumbi, Michuki Maina, Jalemba Aluvaala, Archna Gupta, Merrick Zwarenstein, Newton Opiyo

**Affiliations:** KEMRI-Wellcome Trust Research Programme, P.O. Box 43640, Nairobi, 00100 Kenya; Nuffield Department of Medicine, University of Oxford, Oxford, UK; Ministry of Health, Nairobi, Kenya; Department of Paediatrics and Child Health, University of Nairobi, Nairobi, Kenya; Centre for Studies in Family Medicine, Schulich School of Medicine & Dentistry, Western University, Western Centre for Public Health and Family Medicine, London, Canada

## Abstract

**Background:**

Pragmatic randomized trials aim to examine the effects of interventions in the full spectrum of patients seen by clinicians who receive routine care. Such trials should be employed in parallel with efforts to implement many interventions which appear promising but where evidence of effectiveness is limited. We illustrate this need taking the case of essential interventions to reduce inpatient neonatal mortality in low and middle income countries (LMIC) but suggest the arguments are applicable in most clinical areas.

**Discussion:**

A set of basic interventions have been defined, based on available evidence, that could substantially reduce early neonatal deaths if successfully implemented at scale within district and sub-district hospitals in LMIC. However, we illustrate that there remain many gaps in the evidence available to guide delivery of many inpatient neonatal interventions, that existing evidence is often from high income settings and that it frequently indicates uncertainty in the magnitude or even direction of estimates of effect. Furthermore generalizing results to LMIC where conditions include very high patient staff ratios, absence of even basic technologies, and a reliance on largely empiric management is problematic. Where there is such uncertainty over the effectiveness of interventions in different contexts or in the broad populations who might receive the intervention in routine care settings pragmatic trials that preserve internal validity while promoting external validity should be increasingly employed.

**Summary:**

Many interventions are introduced without adequate evidence of their effectiveness in the routine settings to which they are introduced. Global efforts are needed to support pragmatic research to establish the effectiveness in routine care of many interventions intended to reduce mortality or morbidity in LMIC. Such research should be seen as complementary to efforts to optimize implementation.

## Background

Pragmatic randomized trials (either individual or cluster) focus on effectiveness and are conducted under conditions which closely mimic the routine settings into which the intervention will be introduced [1]. They aim to be rigorous retaining internal validity but promoting external validity. The degree to which a trial is designed to be pragmatic can be considered with respect to nine domains (eligibility, recruitment, setting organization, flexibility in delivery and adherence, follow-up, primary outcome and primary analysis) and the importance of such trials in high income settings has recently been highlighted [2]. The increased attention to pragmatic trials recognizes that many explanatory trials with strict inclusion criteria conducted under idealized conditions may not represent the effectiveness of interventions in the contexts in which they are deployed and the broad patient populations that receive them. There may be particular challenges of applying their results from high income settings to low and middle income settings (LMIC). Pragmatic trials aim to be contextually sensitive and tackle questions and outcomes that are important to policy makers, practitioners, and patients. They are typically conducted in multiple routine care settings with interventions compared with the existing standard of care. Studies should include all patients encountered in typical practice who should be given care in the same way as non-trial patients, they receive no more than usual levels of effort to promote their adherence to regimens, and they are followed up with no more than the intensity expected in usual care [1]. They are therefore designed to have low impact on usual care, for easier integration into workflow. While they may be larger than efficacy trials they tend to be cheaper as the data collection effort is minimized. Rather than addressing an explanatory (biological or mechanistic) question such research therefore addresses effectiveness-in-context questions with generalizability to similar contexts.

To illustrate why more such trials are needed to support improved facility based care in LMIC we focus on interventions that may be used as part of inpatient neonatal care. The most recent global burden of disease estimates suggest that 44 % of the 6 · 3 million deaths under 5 years in 2013 occurred in the neonatal period, a disproportionate number in LMIC. The three leading causes of neonatal death are preterm birth complications, intra-partum events (leading to hypoxic ischaemic encephalopathy formerly termed birth asphyxia) and severe infection [3]. In addition, term but small for gestational age babies are at increased risk of neonatal death while pathological neonatal jaundice is a significant contributor to disability. To reduce neonatal mortality and morbidity most effectively Bhutta and colleagues identified sets of high impact interventions to be delivered pre-partum or after birth [4]. These authors suggested that ‘the greatest effect would come from a focus on the care of small and ill neonates, which has been neglected to date’, further stating that ‘much of this effect is potentially achievable through newborn care services in sub-district and district level hospitals.’ It is for this reason that we focus here on inpatient neonatal care. We acknowledge that this focus excludes many important aspects of care that support newborn survival. We also acknowledge that many important pragmatic trials already inform recommendations for newborn care in LMIC, particularly trials aimed at community based or primary care (for example [5–9] and the individual trials contributing for example to reviews [10, 11]). However, our purpose is not to provide a comprehensive overview of the state of knowledge in newborn care or even in a particular field. Instead we use the example of inpatient newborn care to illustrate that a close look at any field is likely to demonstrate that much greater attention should be paid to the conduct of pragmatic trials.

We make the case for more trials as currently the challenge of reducing neonatal mortality could be seen simply as one of solving problems of intervention delivery at scale in LMIC by improving coverage [4, 12]. Coverage with quality care is critical. But as we consider and test which delivery strategies might work best in which contexts, what of the interventions themselves? How certain are we of their effectiveness and for whom? In the management of the sick newborn child in resource limited settings treatment options available are relatively few, and even apparently simple aspects of management (how best to give oxygen, fluid, or feeds or treat and prevent hypoglycaemia or hypothermia) are often not supported by high quality evidence with often very little good, contextually generated evidence. Improvement in outcomes in LMIC requires better knowledge of when, how, to whom and by whom such interventions are delivered as part of learning how best to employ and implement advancing levels of care in settings with limited access to organ-supportive technologies/interventions. We use the case of interventions that are intended to improve inpatient newborn survival to illustrate this need for pragmatic trials to address these questions. As a large number of neonatal deaths occur in the very first days of life a focus on immediate outcomes in such trials would appear justified. We suggest however that close scrutiny in many clinical areas would suggest similar needs for pragmatic trials.

## Discussion

We contend that for many interventions (not just these in inpatient neonatal care) that further evidence of effectiveness when they are provided within routine settings in LMIC is needed and that a major pragmatic effectiveness research agenda needs to run in parallel with efforts to improve service delivery and strengthen health systems. We summarise what is known from randomised controlled trials about a number of neonatal interventions to be delivered in small hospitals in Table 1, indicate how much data are available from LMIC, and discuss some in more detail below.

**Table 1:** Evidence on interventions to improve inpatient neonatal morbidity and mortality in a selection of existing systematic reviews

Intervention	Availability of evidence in systematic reviews		
	Total no. of trials (Trials in LIC)	Total no. of infants (Infants in LIC)	Review authors conclusions and comments
Chlorhexidine skin or cord care for prevention of mortality and infections in neonates born in the community and in facility births	4 (4) cluster RCT in community	72909 (72909)	There is high-quality evidence that chlorhexidine skin or cord care in the community setting results in a 50 % reduction in the incidence of omphalitis and a 12 % reduction in neonatal mortality. There is some uncertainty as to the effect of chlorhexidine applied to the umbilical cords of newborns in hospital settings on neonatal mortality [11].
	4 (1) RCT in hospital settings	1451 (146)	
Effect of plastic wrappers to prevent mortality in preterm and/or low birthweight infants or to prevent hypothermia on admission to newborn units	4 (0) RCT for mortality	264 (0)	Plastic wraps or bags lead to higher temperatures on admission to neonatal units and less hypothermia. However, the small numbers of infants and studies and the absence of long-term follow-up mean that firm recommendations for clinical practice cannot be given [18, 32]. A more recent trial of 104 babies in Malawi suggested plastic wrappers prevent hypothermia but there was no effect on mortality [32].
	2 (0) RCT for hypothermia	152 (0)	
Total volume of water (fluid) intake in early life in preterm infants and optimum initial glucose supply rates when intravenous fluids must be used	5 (0) RCT	582 (0)	Restricted water intake significantly increases postnatal weight loss but significantly reduces the risks of patent ductus arteriosus and necrotizing enterocolitis. With restricted water intake, there were trends toward increased risk of dehydration and reduced risks of bronchopulmonary dysplasia, intracranial hemorrhage, and death [21].
Optimum initial glucose supply rates when intravenous fluids must be used	2 (0) RCT	51 (0)	There is insufficient evidence from trials comparing lower with higher glucose infusion rates to inform clinical practice [20].
Early trophic feeding versus enteral fasting for very preterm or very low birth weight infants (effect on mortality)	8 (0) RCT	558 (0)	Early trophic feeding is associated with a non-significant reduction in mortality 0.66 [0.41, 1.07] [33]
Delayed introduction of progressive enteral feeds and slow or rapid advancement of feeds to prevent necrotising enterocolitis (NEC) in very low birth weight infants	8 (0) RCT Delayed introduction	1092 (0)	Delaying introduction of progressive enteral feeds to four days or more after birth did not reduce the risk of NEC (typical RR 0.93, 95 % CI 0.64 to 1.34; 8 trials; 1092 infants) [22]. There were no statistically significant effects on the risk of NEC of fast rates of feed advancement (typical risk ratio (RR) 0.96, 95 % confidence interval (CI) 0.55 to 1.70) or all-cause mortality (typical RR 1.57, 95 % CI 0.92 to 2.70) [23].
	6 (3) RCT Rate of advancement	618 (183)	
Effect of nasogastric or orogastric feeding tubes on time to establish full enteral feeding in preterms who have respiratory distress	1 (0) RCT	46 (0)	There are insufficient data available to inform practice [25].
Whether continuous, slow feeding is safer or more effective than bolus feeding in preterm infants	5 (0)	424 (0)	There were no differences in time to achieve full enteral feeds in studies comparing continuous nasogastric versus intermittent bolus nasogastric milk feedings (WMD 2 days, 95 % CI -0.3 to 3.9) [24].
Alternatives to phenobarbitone as first line treatment for convulsing newborns and optimal second line treatment for continued convulsions in a newborn after receiving phenobarbitone	1 (0) RCT and 5 (1) observational studies for first line treatment	59 (0) in RCT and 717 (101) in observational studies	There is very low quality evidence comparing phenobarbitone with phenytoin as first line treatment and very low quality evidence informing decisions on second line treatment. There is a similar deficit in evidence to inform duration of therapy, whether to use anticonvulsant prophylaxis in babies with hypoxic ischaemic encephalopathy and strategies to stop treatment. There is no evidence to inform decisions on empiric treatment for hypoglycaemia in convulsing newborns and so testing blood glucose in convulsing newborns is recommended prior to starting anticonvulsants [26].
	1 (0) RCT and 1 (0) observational study for second line treatment	8 (0) in RCT and 45 (0) in observational study	
Kangaroo Mother Care (KMC)	8 (7) RCT; 1 (1) of these RCT in babies pre-stabilisation	1736 (1676); 123 in a trial of babies pre-stabilisation	The evidence from this updated review supports the use of KMC in LBW infants as an alternative to conventional neonatal care mainly in resource-limited settings. Further information is required concerning effectiveness and safety of early onset continuous KMC in unstabilized or relatively stabilized LBW infants, long term neurodevelopmental outcomes, and costs of care [10].
Topical emollient therapy	8 (2) RCT	2086 (535)	There is no clear evidence that use of emollient therapy prevents invasive infection (RR 1.13, 95 % CI 0.97-1.31) or mortality (RR 0.87 95 % CI 0.75-1.03) [27].
Safety and efficacy of CPAP (particularly bubble CPAP) as a primary respiratory support to improve survival, compared with standard oxygen therapy *in low and middle income countries*.	(4) RCT plus (5) case control and cohort studies	(450) in RCT and (1408) in observational studies	There is evidence that CPAP is safe and may reduce the need for mechanical ventilation. However more studies are recommended to compare CPAP and existing standard oxygen therapy in low and middle income countries [28].
Comparisons of alternative empiric antibiotic regimens for early onset neonatal sepsis	2 (0) RCT	127 (0)	There is no evidence from randomised trials to suggest that any antibiotic regimen may be better than any other in the treatment of presumed early neonatal sepsis. More studies are needed to resolve this issue [16]. There is inadequate evidence from randomised trials in favour of any particular antibiotic regimen for the treatment of suspected late onset neonatal sepsis [15]. There was insufficient evidence to recommend a particular antibiotic regimen for the treatment of NEC [17]. In guidance WHO updated in 2012 on empiric treatment of neonatal sepsis the quality of evidence was judged to be low [14].
Comparisons of alternative empiric antibiotic regimens for late onset neonatal sepsis	1 (0)	28 (0)	
Comparisons of alternative empiric antibiotic regimens for necrotising enterocolitis	2 (0)	62 (0)	
Use of prophylactic phototherapy to prevent cerebral palsy or all-cause mortality in preterm infants (<37 weeks or <2500 g)	2 (0) RCT Cerebral palsy	756 (0)	Prophylactic phototherapy helps to maintain a lower serum bilirubin concentration and may have an effect on the rate of exchange transfusion and the risk of neurodevelopmental impairment. However, further well-designed studies are needed to determine the efficacy and safety of prophylactic phototherapy on long-term outcomes including neurodevelopmental outcomes [30].
	4 (1) RCT Mortality	3044 (50)	
Single versus double volume exchange transfusion in treatment of ABO incompatibility	1 (0)	20 (0)	There was insufficient evidence to support or refute the use of single volume exchange transfusion as opposed to double volume exchange transfusion in jaundiced newborns [29].

When a mother goes into pre-term labour it has been conclusively shown in high income settings that giving her steroids before the birth reduces the severity of lung disease the baby may have as a result of their prematurity. However, a recent large, pragmatic trial has shown that the contextual challenges of giving this intervention at the right time in settings where the duration of the pregnancy is not accurately known resulted in no overall benefit and potential harm to mothers and babies [5]. A major effort is now therefore underway to try and establish how best to utilize this intervention in practice in LMIC more than two decades after its efficacy was established.

Around the time of birth itself two interventions are aimed at preventing early and serious infections of the baby. Cleaning the umbilical cord with chlorhexidine from birth for one week has been shown to save lives when babies are born at home, often without a skilled attendant, in low-income settings [11]. Although it is now proposed the same cord cleaning should be used for babies born in health facilities, where clean birth practices may limit risks of infection, there is little evidence to support its use in this setting. Babies are sometimes born with what are felt to be risk factors for early infection. These include prolonged, pre-labour rupture of membranes for term deliveries, maternal fever or clinically diagnosed chorioamnionitis or preterm birth [13]. Concern over such risk factors prompted the World Health Organisation to suggest that presumptive antibiotics be given to babies exposed to these risks in LMIC on the grounds that there is a higher absolute risk of infection, because these settings lack sufficient well-trained staff to regularly review at risk babies for signs of illness and because clinicians do not have access to diagnostic tests to guide their decision making if immediate antibiotic prophylaxis is withheld. However, it has been acknowledged by WHO and some high income countries that any recommendations are based largely on expert panel opinion rather than evidence [13, 14]. In an era of concern over rising anti-microbial resistance understanding whether prophylactic antibiotics can be safely withheld in this population seems an important question.

In the first days and weeks of life newborns are at high risk of infection. Antibiotic treatment of suspected neonatal sepsis would appear an obvious essential intervention. Recent large trials have tested primary care approaches to treatment of babies with clinical signs suggesting low risk of infection [6–8]. For those with a clinical presentation suggesting more serious disease very few babies have been included in trials comparing the standard treatment (penicillin or ampicillin with gentamicin) with alternative regimens [15, 16] although CSF penetration of gentamicin is relatively poor and therapeutic drug monitoring to prevent toxicity is not available routinely in most LMIC. Similarly data are largely absent to guide appropriate choice of therapy for necrotizing enterocolitis [17] or to guide duration of therapy where laboratory diagnostics (culture and inflammatory markers) are not available.

Once a baby is born an immediate concern is keeping it warm. Two specific interventions, plastic wraps for very small preterm babies and kangaroo mother care can keep a baby warm and are included in a proposed set of essential interventions aimed at preventing morbidity and mortality in pre-term infants [4]. Evidence is limited but does suggest plastic wraps preserve babies’ temperature initially but there are few data from LMIC and the effect on more substantive outcomes is unknown [18]. For Kangaroo mother care systematic review suggests it is associated with a 40 % reduction in mortality when used in low birthweight babies [10]. However, 16 studies (8 RCT) initiated KMC after babies had stabilized (and therefore after most mortality has occurred) and only one study of 123 babies initiated KMC within 24 h of birth when they were ‘unstable’. Although this study in unstable preterms reported a major reduction in mortality half of eligible babies were not recruited, a substantial number of deaths and the major difference between trial arms occurred within 12 h of birth, and there was potential baseline imbalance [19]. Questions remain therefore on how early to start KMC in unstable, preterm babies and what its impact might be in practice in this group that accounts for most preterm mortality.

Babies born preterm or small for gestational age often require ongoing, facility based care. Providing adequate fluid and nutrient intake is clearly important to short term survival and long term health and development, ‘feeding’ is therefore an essential intervention. But how do we provide optimal fluid and nutrient intake in LMIC? In high income settings the smallest babies who have the highest mortality risk are frequently only given intravenous fluids in the first days of life. This may be followed by very gradual introduction of enteral feeds over a period of days during which they also receive intravenous nutrition. In high income settings one nurse may be caring for 2 to 4 babies depending on their illness severity and monitoring of blood glucose, respiratory effort, oxygenation and wider assessment of clinical well-being are frequent, but the nurse ratios and ability to monitor are very different in LMIC settings. Is the evidence then relevant, given that the standard of care is likely to be much lower due to reduced staffing?

In the first days of life when fluids may have to be given intravenously there is uncertainty over which glucose infusion rate is optimal for preventing hypo or hyperglycaemia [20] and uncertainty of the balance of risks and benefits from liberal or more restricted fluid intake [21] in preterm infants. Key questions also remain over how best to introduce milk (enteral) feeds. Evidence from high income settings is sparse but suggests introducing milk feeds before the 4^th^ day of life and advancing feed volumes quickly each have no more risk than delaying their introduction or advancing feed volumes more slowly [22, 23]. If one starts feeds evidence provides little guidance for whether nasogastric tubes that may obstruct unassisted ventilation more than orogastric tubes or bolus or continuous feeding are advantageous [24, 25]. While these limitations of the existing evidence are relevant to all settings the context for providing nutritional support in many LMIC may be very different. Nurses care for many more babies, blood glucose testing may be impossible or performed very rarely, electrical monitoring of physiological status, electrically powered pumps to deliver low volumes of fluids accurately and intravenous nutrition are all absent. The latter aspects in particular raise major questions. Is it better to initiate feeds immediately after birth and rapidly escalate volumes, potentially risking respiratory deterioration or necrotizing enterocolitis, or delay and provide inadequate calorie intake because intravenous nutrition is not available? Is it safe to give intravenous fluids at all when only adult fluid giving sets are available that make accurate regulation of infused volumes to preterm babies extremely difficult, especially when nurses are so few?

A similar paucity of evidence of effectiveness affects selection of anticonvulsants for prophylaxis and treatment of convulsions common after severe asphyxia [26], the use of topical emollients to prevent sepsis [27], the use of continuous positive airways pressure devices for respiratory distress [28] and how best to manage jaundice [29, 30].

## Conclusions

Improving health outcomes will require timely delivery of interventions that are acceptable to and effective for all those affected by illness and feasible and affordable for delivery in LMIC contexts. To ensure investments are made in delivering the right interventions we need appropriate evidence of real world effectiveness obtained directly in relevant contexts. Such studies should underlie and can be linked to efforts to deliver appropriate bundles of care within local health systems that may be the focus of implementation research. However, focusing just on implementation strategies may be inadequate if the effectiveness of clinical interventions is in doubt and many of the questions we highlight are not reflected in research priority setting exercises [31] despite the paucity of evidence being highlighted by an increasing number of systematic reviews. Indeed in the World Health Organisation’s recent technical update to their successful Pocket Book of Hospital Care for Children 40 of the 54 (74 %) recommendations to guide essential paediatric and neonatal practice were made on the basis of low or very low certainty in evidence of effects. Addressing these gaps in primary evidence through pragmatic trials should be a priority on the global research agenda.

Delivering on an agenda for pragmatic trials will require strategic attention. Gathering pace over the last two decades there have been considerable efforts to support conduct of and training on RCT tackling efficacy questions in LMIC – although this agenda is unfinished. Research ethics committees in LMIC often now have clear, internationally consistent guidance on regulation of such trials and LMIC research institutes may have well developed trial sites. However, these institutions may be much less familiar with pragmatic designs that take place within routine settings where the aim is not to transform and tightly regulate the standard of care. They may also be less familiar with or have less well developed policies on consent where routine data or cluster randomization are employed. As pragmatic research takes place within the health system a major issue going forward will also be building much closer links with policy makers, managers, health care staff, those building health information systems and communities so that they both understand and contribute to the research agenda.

A fundamental technical asset that would facilitate pragmatic research is for much improved health information systems, a need shared with those making efforts to monitor impact of new delivery strategies or system strengthening efforts. But most important we need to produce a new generation of scientists and potentially new institutions that can undertake pragmatic research in LMIC. These scientists should have expertise in RCT design and conduct, be embedded in the realities of routine care, and also be conversant with and linked to the worlds of implementation science and health systems research. This would enable the use of multi-method research programs, and ensure that the questions tackled and outcomes studied and the results obtained are useful for policy makers, clinicians in these settings, and ultimately, for patients.
